# A commentary on ‘The role of cytoreductive nephrectomy in metastatic renal cell carcinoma in the targeted therapy and immunological therapy era: a systematic review and meta-analysis’

**DOI:** 10.1097/JS9.0000000000001254

**Published:** 2024-03-11

**Authors:** Liyao Chen, Yiqiang Liu, Jierui Yang, Aihua Guo, Gaoyang Zhou

**Affiliations:** a Department of Nephrology, The Second People’s Hospital of Jiangyou, Jiangyou, Sichuan; b School of Medical Technology, Xi’an Medical College, Shaanxi; c Department of Neurosurgery, Second Hospital of the Air Force Medical University (Tangdu Hospital), Xi’an, Shaanxi; d Department of Nephrology, Affiliated Hospital of Shaanxi University of Chinese Medicine, Xianyang, Shaanxi, People’s Republic of China

*Dear Editor*,

It was with great interest that we read the article published in the *International Journal of Surgery*. Chen *et al*.^1^ conducted a systematic review and meta-analysis of patients with distant metastatic renal cell carcinoma (mRCC) who had undergone cytoreductive nephrectomy with or without targeted therapy (TT) and immunological therapy. In individuals with mRCC, it appears that cytoreductive nephrectomy (CN) was linked to a higher survival rate than no CN.

CN is the removal of the kidney and primary tumor in patients with mRCC, and its effect on mRCC is still debated in the TT and immune therapy eras^2,3^. The majority of evidence from modern IO (immuno-oncology) is inexorably restricted to collective database reviews and retrospective studies, the majority of which yield positive outcomes. Findings from the CARMENA and SURTIME trials support the logical conclusion that upfront chemotherapy (CN) in synchronous clear-cell multicenter RCC patients carries a significant risk of postponing systemic treatment for patients who could otherwise worsen.

This article has numerous benefits and serves as inspiration. Initially, this study had a big sample size, especially for the OS (overall survival) analysis. Second, in order to obtain precise risk estimates, the quality of cohort studies and randomized control studies is assessed separately using the ROBIN-1 and RoB 2 tools. Finally, sensitivity analysis was performed to assess the dependability of the pooled results. Overall, hazard ratio estimates for these survival outcomes were shown to be steady and dependable in the meta-analysis results, according to the leave-one-out test of sensitivity analysis. However, there were still a lot of restrictions. First, three randomized control studies and 27 cohort studies were included in this meta-analysis. It is preferable to conduct subgroup analyses using different types of study after a comprehensive analysis, which may lessen the huge heterogeneity of the included studies. Second, two of the randomized control studies seem to come from the same clinical trial, which just has different ways of stratifying patients by the number of risk factors^4,5^. One of the studies^5^ hasn’t used meta-analysis but system review; it may not be inappropriate to say that it has included three randomized control studies in this meta-analysis. To verify this result, more thorough and multicenter research is needed. In addition, additional prospective studies, clinical trials, and analysis of indications and risk factors in different patient subgroups are needed to determine the optimal course of treatment.

Future trials are required to assess the advantages and limitations of CN in the immunotherapy era, customize the treatment regimen, and identify the patients who are most likely to benefit from surgical treatments as systemic therapies for mRCC continue to advance.

## Ethical approval

This manuscript is a comment. Don’t need ethical approval.

## Consent

This manuscript is a comment. Don’t need patient consent.

## Author contribution

L.C. and Y.L.: study concept or design, data collection, data analysis or interpretation, and writing the paper; J.Y. and A.G.: data collection; G.Z.: study concept or design, writing, and revising the paper.

## Conflicts of interest disclosure

The author declares no conflicts of interest.

## Research registration unique identifying number (UIN)

This manuscript is a comment. It does not need UIN.

## Guarantor

Gaoyang Zhou.

## Data availability statement

This manuscript is a comment. Don’t need a Data availability statement. However, all the data from the current study are publicly available.

## Provenance and peer review

This manuscript is a comment without being invited.
